# A key to the flat grass scale genus *Nipponaclerda* (Hemiptera, Coccomorpha, Aclerdidae)

**DOI:** 10.3897/zookeys.862.35294

**Published:** 2019-07-09

**Authors:** Scott A. Schneider

**Affiliations:** 1 USDA, Agricultural Research Service, Henry A. Wallace Beltsville Agricultural Research Center, Systematic Entomology Laboratory, Building 005 – Room 004, 10300 Baltimore Avenue, Beltsville, MD, 20705, USA USDA, Agricultural Research Service Beltsville United States of America

**Keywords:** Identification, invasive species, Louisiana, pest, scale insect, *Phragmitesaustralis*

## Abstract

The flat grass scale genus *Nipponaclerda* comprises four species, native to Central and East Asia. *Nipponaclerdabiwakoensis* has been introduced to the United States and is considered a serious pest of *Phragmitesaustralis*, the common reed. Heavy infestations of *N.biwakoensis* in coastal marshes of Louisiana have coincided with extensive die-off of reeds. In this article, dichotomous identification keys to the genera of Aclerdidae and to the species of *Nipponaclerda* are provided, allowing for accurate identification of species found in the native and invasive range.

## Introduction

The genus of flat grass scale insects, *Nipponaclerda* McConnell (Hemiptera, Coccomorpha, Aclerdidae), comprises four species native to Central and East Asia, one of which has been introduced to the United States. This small group is receiving attention due to the recent discovery of *Nipponaclerdabiwakoensis* (Kuwana) (the roseau cane scale) in Louisiana, U.S.A. (Knight et al. 2018). The detection of invasive populations of *N.biwakoensis* in Louisiana coincided with the discovery of extensive die-off of *Phragmitesaustralis* (Cav.) Trin. Ex Steud. (i.e., common reed, roseau cane). In 2016, surveyors found large stands of dead or dying *P.australis* in Plaquemines Parish, Louisiana (Knight et al. 2018), part of the Mississippi River Delta (MRD). This sparked concern due to the reeds’ importance to marsh ecosystems and coastal infrastructure. Losses of *Phragmites* in the MRD could have substantial, negative economic repercussions. Roseau cane is the dominant emergent plant in the MRD; it builds, maintains, and protects marsh soils and provides habitat for diverse wildlife. Dense stands of roseau cane help reduce wave action, which shields coastal regions from damaging storm surges and hurricanes. The marshes also shelter a network of shipping channels as well as oil and gas infrastructure in this region.

Presently, *N.biwakoensis* is the only member of this genus to be considered a pest. Heavy infestations in Louisiana reached over 2,000 individuals per stem in 2016–2017 (Knight et al. 2018). In China it is considered a pest of *P.australis*, which is cultivated for the paper industry (Brix et al. 2014). Natural enemies help keep populations at low abundance in the native range, and in Japan and China *N.biwakoensis* served as a model for studies about parasitism by hymenopterans and predation from birds (Kaneko 1995; 2004; 2005; Xiong et al. 2010).

Aclerdids feed primarily on grasses (Poaceae), which include some of the most important commodities such as maize, rice, wheat, and sugarcane. But damaging infestations of aclerdids are uncommon on commodities (McConnell 1954), which explains why aclerdids have received minimal attention in the taxonomic literature, where efforts skew toward larger groups with more significant agricultural impact. Thus, Aclerdidae is an especially difficult group to identify to species. The only key ever published to differentiate between species of *Nipponaclerda* was by Borchsenius (1960), which can be used to separate *N.biwakoensis* from *N.turanica* (Archangelskaya) (reproduced in Wang 1994). Considering the potential economic impact of *N.biwakoensis*, a history of introduction to the United States, and the possibility of discovering additional species, it is important to have a tool that allows for accurate identification of species in this genus.

## Materials and methods

Specimens of *N.biwakoensis* collected from Louisiana and Texas were slide-mounted following the protocol of the Systematic Entomology Laboratory (USDA, ARS) at Beltsville, Maryland (http://www.ars.usda.gov/Main/docs.htm?docid=9832). DNA extractions were performed on some specimens prior to slide-mounting (see Knight et al. 2018); others were prepared without having an extraction performed. Specimens are deposited in the United States National Museum of Natural History’s Collection of Coccomorpha, housed in Beltsville, Maryland. Observations were made using a Zeiss Axio Imager.M2 (Carl Zeiss Inc., Thornwood, NY, U.S.A.). Diagnostic information for species of *Nipponaclerda* was drawn from published descriptions and illustrations (Borchsenius 1960; Kuwana 1907; McConnell 1954; Wang and Zhang 1994; Zhang 1998).

**Institutional abbreviations**

**CDFA**California State Collection of Arthropods at California Department of Food and Agriculture

**USNM**The Smithsonian Institute, United States National Museum of Natural History, Entomology Collection, Coccomorpha

## Taxonomy

### Nipponaclerda

Taxon classificationAnimaliaHemipteraAclerdidae

McConnell, 1954

Nipponaclerda McConnell 1954: 107.

#### Type species.

*Aclerdabiwakoensis* Kuwana by monotypy and original designation.

#### Comments.

*Nipponaclerda* are classified within the subfamily Aclerdinae Cockerell (Hodgson and Millar 2002), with *Aclerda* Signoret and *Lecanaclerda* Hodgson & Millar. Members of this genus are similar to species of *Aclerda* but can be distinguished in the adult female stage by the poor development of anal ring setae, which are few in number, shorter than the anal plate, and never extend beyond the body apex (Borchsenius 1960). In adult females of *Aclerda*, these setae are numerous, long, and usually protrude beyond the posterior body margin; however, these setae fail to extend beyond the margin in *A.pasquieri* Balachowsky, *A.sinaloaensis* McConnell, and *A.subterranea* Signoret. Additionally, dorsal conical pores (referred to as invaginated setae in McConnell 1954) can be found in species of *Aclerda* but are absent from *Nipponaclerda*. There are no discernable differences between the immature instars of these two genera but adult males of *Nipponaclerda* can be differentiated by the encircling marginal setae, absence of dorsal invaginated conical pores, and reduction in the number of anal ring setae (McConnell 1954). The adult male stage has only been described for *N.biwakoensis*.

The list of host genera reported in ScaleNet (http://scalenet.info/; last accessed 19 March 2019) for *Nipponaclerda* species includes: *Agropyron*, *Bambusa*, *Fargesia*, *Phragmites*, *Sorghum* (Poaceae), and *Juncus* (Juncaceae) (Borchsenius 1960; García Morales et al. 2016; McConnell 1954; Wang 1994; Wang and Zhang 1994; Zhang 1998).

##### Species list

*Nipponaclerdabiwakoensis* (Kuwana, 1907: 187)

*Nipponaclerdaleptodermis* Wang & Zhang, 1994: 94

*Nipponaclerdatriumpha* Zhang, 1998: 7

*Nipponaclerdaturanica* (Borchsenius, 1950: 156)

##### Key to genera of Aclerdidae based on adult females

**Table:** 

1	Legs and antennae fully developed; pregenital disc-pores present across abdominal segments medially	***Lecanaclerda***
–	Legs and antennae absent or very reduced; pregenital disc-pores absent	**2**
2	Caudal region of abdomen with sclerotized cone; anal cleft absent; most abundant dorsal tubular duct bilocular	**3**
–	Caudal region of abdomen without sclerotized cone; anal cleft clearly present; most abundant dorsal tubular duct without internal divisions or loculi	**4**
3	Thorax with pair of sclerotized brachial plates extending laterally onto dorsum from near spiracles	***Kwazulaclerda***
–	Thorax without pair of sclerotized brachial plates extending laterally onto dorsum from near spiracles	***Rhodesaclerda***
4	Anal ring usually bearing about 10–20 anal ring setae, each longer than length of anal plate, often extending beyond posterior body margin; dorsal conical pores (invaginated setae of McConnell 1954) present	***Aclerda***
–	Anal ring bearing 2 groups of 3–5 setae, each much shorter than length of anal plate, or anal ring setae inconspicuous, never extending beyond body margin; dorsal conical pores (invaginated setae of McConnell 1954) absent	***Nipponaclerda***

##### Key to *Nipponaclerda* based on adult females

**Table:** 

1	Marginal tuberculate setae present; few multilocular disc pores associated with spiracles only; macrotubular ducts present	**2**
–	Marginal tuberculate setae absent; numerous multilocular disc pores arranged in submarginal ring and associated with spiracles; macrotubular ducts absent	**3**
2	Marginal tuberculate setae arranged in continuous unbroken ring; multilocular disc pores variable in number, ranging from 4–20 just anterior to spiracles, rarely absent; microtubular ducts at anterior end of body numerous, arranged in 6–10 irregular rows	***N.biwakoensis* (Kuwana)**
–	Marginal tuberculate setae interrupted at anal cleft, not forming continuous unbroken ring around margin; multilocular disc pores numbering greater than 20 just anterior to spiracles; microtubular ducts at anterior end of body few, scattered, arranged in 1–4 irregular rows	***N.turanica* (Borchsenius)**
3	Vestigial legs present on mesothorax between anterior and posterior spiracles, represented by small tubercle with approximately 7 short setae; atrium of spiracles relatively small with about 2 rows of multilocular disc pores; submarginal patch of microtubular ducts present on dorsum of head	***N.triumpha* Zhang**
–	Vestigial legs entirely absent; atrium of spiracles relatively large with approximately 4 rows of multilocular disc pores; microtubular ducts absent from dorsum of head	***N.leptodermis* Wang & Zhang**

### Nipponaclerda biwakoensis

Taxon classificationAnimaliaHemipteraAclerdidae

(Kuwana, 1907)

#### Material examined.

UNITED STATES • 11 ♀; Louisiana, Plaquemines Parish, Venice, West Bay South End; 29°7.5'N, 89°17.2'W; 1 March 2017; R. Diaz leg.; *Phragmitesaustralis*; USNM • 2 immatures; same collection data as for preceding; 1 March 2017; R. Diaz leg.; *Phragmitesaustralis*; USNM • 2 ♀; Texas, Jefferson County, Port Arthur, near J.D. Murphree WMA; 29°53.2'N, 94°2.2'W; 11 July 2018; I.A. Knight leg.; *Phragmitesaustralis*; USNM • 1 ♀; quarantine interception at California, San Diego, originating from Japan; 2 April 1959; L. Widman leg.; *Phragmites* sp. or rush; CDFA • 3 ♀; quarantine interception at California, Los Angeles, San Pedro, originating from Japan; 22 March 1960; M.F. Brown, Jr. leg.; *Phragmitescommunis* (=*australis*); USNM • 1 ♀; quarantine interception at California, Stockton, originating from Japan; 25 May 1961; R.E. DeVol leg.; *Phragmitescommunis* (= *australis*); USNM • same collection data as for preceding; 3 ♀; 25 May 1961; R.E. DeVol leg.; *Phragmitescommunis* (=*australis*); CDFA • 2 ♀; quarantine interception at Hawaii, originating from Japan; 25 March 1960; L. Chilson leg.; *Phragmitescommunis* (=*australis*) stems; USNM. CHINA • 2 ♀; Taiwan, Taichung County; 24°19.2'N, 12°33.5'E; 22 August 2018; S-G. Syu and J-L. Jhu leg.; *Phragmitesaustralis*; USNM • 3 ♀; Hong Kong Special Administrative Region, Tin Shui Wai, Hong Kong Wetland Park, fresh water marsh; 22°28.1'N, 11°0.4'E; 7 August 2018; B. Brown leg.; *Phragmitesaustralis*; USNM. JAPAN • 2 ♀; Fukuoka, Nishi-Ku, Motooka; 33°35.5'N, 13°13.9'E; 25 October 2018; H. Tanaka leg.; *Phragmitesaustralis*; USNM • 3 ♀, syntype; Omi; August 1902; S.I. Kuwana leg.; on rush; USNM • 5 ♀, syntype; Tokio [Tokyo]; 16 April 1906; S.I. Kuwana leg.; *Phragmitescommunis* (= *australis*); USNM • 4 immatures; same collection data as for preceding • 1 ♀; Tokyo; 18 October 1953; R. Takahashi leg.; *Phragmites*; USNM.

#### Notes.

Adult females of *N.biwakoensis* are similar in appearance to *N.turanica* but the two can be easily distinguished by the traits mentioned above, and in addition by the pattern of sclerotization and by the types of setae located on the anal plate and posterior margin. The posterior end is heavily sclerotized in *N.turanica*, whereas in *N.biwakoensis*, moderate sclerotization is more evenly distributed along the marginal rim, becoming more heavily sclerotized in mature females. *N.biwakoensis* possesses some tuberculate setae on the anal plate and bears only tuberculate or spine-like setae on the posterior body margin. In contrast, only flagellate setae are present on the anal plates of *N.turanica* and several flagellate setae fall on the posterior body margin. *N.biwakoensis* was well-illustrated by McConnell (1954); additional illustrations were published by Kuwana (1907; 1932) and Wang (1994).

Several natural enemies of *N.biwakoensis* are reported in the literature. The parasitoids *Astymachusjaponicus* Howard, *Boucekielladepressa* Hoffer, *Platencyrtusaclerus* Xu (Hymenoptera: Encyrtidae), and *Aprostocetus* sp. (Hymenoptera: Eulophidae) have been reported from the native range (Kaneko 2004; Xu and Wang 2003), which includes China, Japan, and South Korea (García Morales et al. 2016). Knight et al. (2018) reported *Neastymachusjaponicus* Tachikawa, *B.depressa*, and *Astymachus* sp. (Hymenoptera: Encyrtidae) parasitizing populations in Louisiana, U.S.A.

It is difficult at present to determine the origin and timing of invasion to the United States. Specimens of *N.biwakoensis* were encountered in quarantine interceptions originating from Japan three times in California and once in Hawaii, between 1959 and 1961. But populations have only been collected from the U.S. since 2016 (Knight et al. 2018), suggesting their establishment is a recent event. Infestations have been found in Louisiana and eastern Texas on *Phragmitesaustralis*, which appears to be the primary host for this species. *N.biwakoensis* was also reported on species of *Agropyron* and *Juncus* (Wang 1994), but these host records should be confirmed through further sampling.

## Supplementary Material

XML Treatment for Nipponaclerda

XML Treatment for Nipponaclerda biwakoensis
